# Work-Related Quality of Life, Mental Health, and Female Sexual Well-Being Among Nurses

**DOI:** 10.3390/healthcare14111444

**Published:** 2026-05-23

**Authors:** Panagiota Valetta, Ioanna Dimitriadou, Krystalia Gkouletsa, Aikaterini Toska, Maria Saridi, Anna Mavroforou, Evangelos C. Fradelos

**Affiliations:** 1 Faculty of Medicine, University of Thessaly, 41500 Larissa, Greece; valettabetty@gmail.com (P.V.); amavroforou@uth.gr (A.M.); 2 Laboratory of Clinical Nursing, Department of Nursing, University of Thessaly, 41500 Larissa, Greece; ioadimitriadou@uth.gr (I.D.); gkrystallia@gmail.com (K.G.); ktoska@uth.gr (A.T.); msaridi@uth.gr (M.S.); 3 Department of Nursing, University of Thessaly, 41500 Larissa, Greece

**Keywords:** WRQoL, well-being, mental health, sexual health, female sexual well-being, women healthcare workers

## Abstract

Introduction: The work-related quality of life affects employee satisfaction and organizational effectiveness, with a direct impact on the quality of healthcare. This study aims to investigate the work-related quality of life (WRQoL) among nurses in tertiary healthcare, as perceived by the nurses themselves, in relation to their demographic and professional characteristics. At the same time, it seeks to highlight the way in which the individual dimensions of WRQoL influence their sexual and mental health. Materials and Methods: A descriptive cross-sectional study was conducted in 2023 in a General Hospital in Greece. Data were collected using structured questionnaires assessing sociodemo-graphic and occupational characteristics, WRQoL, mental health (Depression, Anxiety and Stress Scale—DASS-21), and female sexual function (Female Sexual Function Index—FSFI-19). Pearson correlation analysis and multiple linear regression analysis were performed. The regression model was adjusted for age, marital status, number of children, and work experience. Results: The results demonstrated a significant negative association between depression and sexual function (β = −0.388, *p* = 0.029), while stress was positively associated with sexual function (β = 0.371, *p* = 0.038). The overall regression model was statistically significant (*p* = 0.001), explaining 18.6% of the variance in sexual function. Conclusions: The findings highlight the close interrelationship between work-related quality of life, mental health, and sexual function among nurses. Poorer psychological well-being was associated with reduced sexual function, emphasizing the impact of occupational and emotional burden on nurses’ overall health. These results underline the importance of supportive workplace environments and targeted interventions to promote mental and sexual well-being among healthcare professionals.

## 1. Introduction

Health is a fundamental factor for individual well-being and societal development, as it is directly associated with productivity, functionality, and overall quality of life [1]. According to the World Health Organization, health is defined as a state of complete physical, mental, and social well-being and not merely the absence of disease [2]. Within this context, the work environment emerges as a key determinant that multi-dimensionally affects employees’ health, particularly in demanding professional sectors such as healthcare.

Work satisfaction constitutes perhaps one of the most extensively researched variables within organizational literature. According to Locke [3], work satisfaction is defined as a pleasurable or positive emotional state—or conversely, a negative one—resulting from the experiences a person encounters within the workplace. In essence, a person performs a multidimensional evaluation—ranging from favorable to unfavorable—of all the constituent elements of the work environment, including organizational climate, compensation structures, and interpersonal dynamics with colleagues [4]. In the healthcare sector, where professionals are required to manage high workloads, emotionally demanding situations, and a heightened sense of responsibility, work-related quality of life (WRQoL becomes particularly important, as it directly affects not only their performance but also their personal well-being [5].

More specifically, mental health is an integral component of general well-being and is profoundly shaped by the occupational environment. Persistent exposure to systemic stressors—including rotating shifts, understaffing, and high emotional labor—is a primary driver of occupational stress, clinical depression, and burnout syndrome [6,7,8]. These conditions not only affect employees’ functioning in the workplace but also extend to their personal lives, influencing emotional balance, social relationships, and overall quality of life [9]. Work-related stress and burnout are common phenomena among healthcare professionals, with significant consequences at both individual and organizational levels. Chronic psychological burden leads to reduced productivity, lower job satisfaction, and an increased risk of physical and mental disorders [9]. At the same time, disruption of work–life balance exacerbates these negative effects, creating a vicious cycle that hinders the maintenance of well-being [10,11]. Beyond the mental dimension, particular interest has been given to the contribution of work life to sexual well-being, which is closely associated with self-esteem, the quality of interpersonal relationships, and emotional stability [12]. Therefore, any disturbance in mental health may directly affect sexual functioning and satisfaction.

Female healthcare professionals are often exposed to higher levels of occupational stress, emotional exhaustion, and multiple social roles, which may negatively affect both their mental and sexual health. In particular, chronic stress is associated with sexual dysfunction, reduced sexual desire, arousal difficulties, and lower sexual satisfaction [13]. In the workplace, gender expectations, burnout, and lack of work–life balance are associated with reduced subjective well-being and impairments in sexual life.

In recent years, the World Health Organization [14] has emphasized sexual rights, sexual pleasure, and overall sexual well-being, including, among others, the right to healthcare, access to information and education about sexuality, and the free choice of partners. The scientific community now places increasing emphasis on sexual well-being as an integral component of human health, as it is bidirectionally related to mental and physical well-being. Positive sexuality, which includes sexual satisfaction, self-esteem, and sexual self-efficacy, may function as a protective factor in coping with stressors and enhancing overall health [15].

Occupational risk factors, both accidental and non-accidental, represent significant influences on employees’ sexual life. Among healthcare workers, exposure to infectious agents, injuries from sharp objects, high work-related stress, long shifts, and rotating schedules have been associated with reduced sexual desire, the occurrence of sexual dysfunctions, and an overall decline in quality of life [16,17,18]. At the same time, the mental and physical fatigue caused by these conditions affects sexual functioning regardless of biological sex, while gendered social constructions and expectations shape different experiences and perceptions of sexuality, particularly among women [19].

The functioning of the reproductive system differs between sexes; however, both are affected by occupational and psychological factors. Disruption of the sexual response—at any stage of the cycle of desire, arousal, plateau, orgasm, or resolution—may arise from stressors and lead to dysfunctions such as reduced desire, arousal difficulties, or impaired orgasm, negatively affecting quality of personal life [20,21]. A representative example is the study by Feng Ji et al. (2017), which reported adverse findings regarding employment conditions and the sexual life of nurses working in a tertiary hospital in China [22]. Furthermore, studies on sexual health conducted in various regions of the African continent have shown different levels of sexual satisfaction, not only due to cultural and religious backgrounds but also due to participants’ emotional values [23]. A study in Nigeria [24] concluded that married couples experiencing work-related stress reported moderate sexual satisfaction and reduced intimacy.

Additionally, the COVID-19 pandemic highlighted how profoundly occupational pressure can affect the sexual lives of healthcare professionals. Studies reported a decrease in sexual activity, the emergence of sexual dysfunctions, and increased sexual dissatisfaction as a result of anxiety about virus transmission, the management of extreme preventive measures, and fear of death [24].

In conclusion, the literature indicates that the mental and sexual health of healthcare professionals is directly influenced by WRQoL, gender as a social construct, and biological, psychological, and social factors. Understanding these complex interactions is essential for the development of targeted interventions aimed at promoting well-being, job satisfaction, and overall quality of life among employees.

The aim of the study is to investigate the degree of association between the level of mental health, which will be measured using the DASS-21 index, and quality of working life, as well as individual sociodemographic and occupational characteristics. In parallel, the level of sexual health, which will be measured using the FSFI, will also be examined in relation to quality of working life and individual sociodemographic and occupational characteristics.

## 2. Materials and Methods

A cross-sectional descriptive study was conducted between 5 September 2023 and 5 October 2023 at the General Hospital of Thessaloniki “Hippokration,” in order to investigate the WRQoL and its associations with indicators of mental and sexual health, taking into account sociodemographic and occupational factors.

### 2.1. Participants and Field of Study

All adult female nurses working in the hospital setting were eligible to participate in the study. The sample was obtained using a convenience sampling method. Inclusion criteria were being a nurse, aged over 18 years, actively working during the study period, and willingness to participate. Healthcare professionals from other specialties, underage nurses, nursing students, and male nurses were excluded from the study.

A total of 200 questionnaires were distributed. Of these, 129 were returned fully completed and were included in the final analysis. Twelve questionnaires were excluded due to incomplete responses, while 59 individuals stated that they did not wish to participate in the study. Some variables included missing responses; therefore, the total number of observations may vary across variables.

### 2.2. Study Variables

Data were collected using structured questionnaires that had been translated, culturally adapted, and validated in the Greek population in previous studies. In addition, a questionnaire was developed to record sociodemographic and occupational characteristics, tailored to the objectives of the study.

The questionnaire consisted of four sections. The first section concerned sociodemographic and occupational characteristics and included 13 questions regarding age, marital status and children, educational level, annual income, professional experience, department of employment, type of employment contract, intention to change department, working schedule, and whether the participant held a position of responsibility, as well as three questions regarding medication use, health problems, and any past surgical interventions. These variables were collected to explore potential associations with the main study variables.

The second section assessed WRQoL, where higher scores indicate better perceived WRQoL. This instrument includes 24 items rated on a five-point Likert scale and provides six subscales as well as an overall WRQoL index. The Greek version of the instrument was used, which has been translated, culturally adapted, and validated in the Greek population [25], and has also been applied in studies involving healthcare professionals [26]. The scale demonstrates high reliability (Cronbach’s α = 0.85).

The third section assessed mental health status using the Depression Anxiety Stress Scale (DASS-21), which includes 21 items and three subscales: depression, anxiety, and stress. The Greek version was used, which has been translated, culturally adapted, and validated in the Greek population [27], demonstrating high reliability (Cronbach’s α = 0.88) [28].

Finally, the fourth section assessed sexual functioning using the Female Sexual Function Index (FSFI-19), which includes 19 items across six domains (desire, arousal, lubrication, orgasm, satisfaction, and pain) and provides a total score. The Greek version was also used, which has been translated, culturally adapted, and validated by Zachariou et al. (2017), demonstrating high reliability (Cronbach’s α = 0.92) [29].

Higher scores on the respective scales indicate better WRQoL and sexual functioning, whereas higher scores on the DASS-21 indicate increased levels of depression, anxiety, and stress.

### 2.3. Statistical Analysis

Due to the exploratory nature of the study and the number of potential factors in relation to the sample size, independent variables were analyzed both individually and using multiple regression analysis. A multiple linear regression model was conducted with the total FSFI score as the dependent variable. Independent variables included DASS subscales and WRQoL dimensions. The model was adjusted for age, marital status, number of children, and work experience. For variables that did not follow a normal distribution, non-parametric tests were used: the Mann–Whitney U test for comparisons between two groups and the Kruskal–Wallis H test for comparisons among more than two groups. Where Kruskal–Wallis tests were statistically significant, post hoc analyses were performed using the Mann–Whitney U test with Bonferroni correction for *p*-values. Associations between FSFI, DASS-21, and WRQoL variables were examined using Pearson’s correlation coefficient (r).

### 2.4. Ethical Considerations

The study was conducted in accordance with all ethical and research conduct standards and the principles of the Declaration of Helsinki. It was approved by the Scientific Council—Ethics and Bioethics Committee of the hospital (protocol number 35323/1-8-2023, approval date 4 September 2023). Participation was voluntary and required written informed consent. Anonymity and confidentiality of the data were ensured throughout the study, in accordance with the General Data Protection Regulation (EU) 2016/679 (GDPR) and Greek Law 4624/2019. The data were used exclusively for research purposes, with no reference to individual participants or the hospital, and no financial burden was imposed on the institution.

## 3. Results

The sample consisted of 129 female nurses, with a mean age of 41.20 years (SD = 11.18), ranging from 18 to 62 years. The mean professional experience was 15.91 years (SD = 11.10), with a range from 0 to 37 years.

Regarding sociodemographic characteristics, the majority of participants were married or cohabiting (51.9%), while smaller proportions were single (21.7%) or in a relationship (17.8%). In terms of the number of children, nearly half had no children (47.3%), whereas 31.0% had two children. Most participants had a higher education degree (48.1%), and 25.6% held a postgraduate degree.

With respect to occupational characteristics, most nurses worked in intensive care units (27.1%) or medical wards (19.4%), while 72.9% followed a rotating work schedule. Additionally, 53.5% were permanent staff, and 29.5% held a position of responsibility. A considerable proportion of participants (39.5%) reported that they would not mind remaining in the same department for several years, while 27.9% did not wish to leave.

Finally, 36.4% reported having a diagnosed disease, and 42.6% reported a history of surgical intervention, while a very small proportion (3.1%) were receiving postmenopausal hormone therapy. More detailed information regarding the sociodemographic and occupational characteristics of the sample is presented in Table 1.

Table 2 The table below presents the descriptive statistics of the scales used in the study, as well as their internal consistency indices. The internal consistency of the scales was assessed using Cronbach’s alpha coefficient. Values above 0.70 were considered acceptable, in line with established recommendations in the literature [30].

The Female Sexual Function Index (FSFI) is a self-administered tool for assessing female sexual function, which includes six domains: desire, arousal, lubrication, orgasm, satisfaction, and pain. The total score ranges from 2 to 36, with higher scores indicating better sexual function. A cutoff score of ≤26.55 has been proposed to distinguish between women with and without sexual dysfunction. Scores below this threshold are associated with an increased likelihood of sexual dysfunction [31]. The tool has been widely used and validated across different populations.

The Depression Anxiety Stress Scales—21 (DASS-21) is a brief self-report psychometric instrument that assesses three dimensions: depression, anxiety, and stress. It consists of 21 items, and the scores of each subscale are multiplied by two for comparison with the DASS-42 norms. Interpretation is based on severity categories (normal, mild, moderate, severe, extremely severe) [32]. Indicatively, for depression, scores ≥10 indicate at least mild symptomatology; for anxiety, ≥8; and for stress, ≥15. The instrument is dimensional and is not used for diagnostic purposes.

The WRQoL (WRQoL) scale evaluates WRQoL through multiple dimensions such as well-being, job satisfaction, and work-related stress. Items are usually scored on a 1–5 Likert scale, with higher scores indicating better WRQoL. There are no established universal cutoff points for categorizing the results. Interpretation is primarily based on the mean score, subscales, and comparisons with other samples. In research practice, scores below the average (~3) indicate lower quality of life, while higher scores indicate better quality of life [33,34]. The SAW subscale demonstrated low internal consistency (Cronbach’s α = 0.449), which is below the acceptable threshold and should be considered when interpreting related findings.

As presented in Table 3, bivariate analyses indicated that sexual functioning was associated with several mental health and WRQoL variables. Depression showed a significant negative correlation with the total FSFI score (r = −0.211, *p* < 0.05) and a stronger negative association with the sexual satisfaction domain (r = −0.302, *p* < 0.001). Anxiety was not significantly associated with the total FSFI score (r = −0.128, *p* > 0.05), although it showed a weak negative correlation with sexual satisfaction (r = −0.192, *p* < 0.05). Stress was not significantly related to the total FSFI score (r = −0.094, *p* > 0.05), but demonstrated a weak negative association with sexual satisfaction (r = −0.181, *p* < 0.05).

Regarding WRQoL, general well-being (GWB) was positively associated with both the total FSFI score (r = 0.217, *p* < 0.05) and sexual satisfaction (r = 0.308, *p* < 0.001). Additionally, several WRQoL subscales, including home–work interface (r = 0.218, *p* < 0.05), job and career satisfaction (r = 0.192, *p* < 0.05), and working conditions (r = 0.207, *p* < 0.05), showed weak positive correlations with sexual satisfaction, while no significant associations were observed with the total FSFI score.

In the multivariable regression analysis, depression (β = −0.388, *p* = 0.029) and stress (β = 0.371, *p* = 0.038) were identified as statistically significant predictors of the total FSFI score. Depression was negatively associated with sexual function, while stress showed a positive association. Anxiety and all WRQoL dimensions, including general well-being, were not statistically significant predictors (all *p* > 0.05).

These findings indicate that although several variables showed significant bivariate associations with sexual functioning, only depression and stress independently contributed to sexual function in the adjusted regression model.

Multiple linear regression analysis was conducted with the total FSFI score as the dependent variable (Table 4). The independent variables included the DASS subscales (depression, anxiety, stress) and the dimensions of WRQoL, adjusting for age, marital status, number of children, and years of professional experience. Adjustment variables (age, work experience, number of children, and employment status) were included in the model to control for potential confounding effects. Their coefficients are not shown, as they were not the primary variables of interest. The regression model explained 29.8% of the variance (R^2^ = 0.298, adjusted R^2^ = 0.186), with a standard error of the estimate of 7.528. Multicollinearity diagnostics were within acceptable limits (VIF < 5; tolerance > 0.20), indicating no significant multicollinearity among predictors. The results showed that depression (β = −0.388, *p* = 0.029) and stress (β = 0.371, *p* = 0.038) were statistically significant predictors of FSFI scores, while anxiety was not statistically significant. Among the WRQoL dimensions, all variables were non-significant predictors of FSFI scores. Specifically, general well-being showed a positive but non-significant association with sexual function (*p* > 0.05), as did home–work interface, control at work, working conditions, job and career satisfaction, and stress at work (all *p* > 0.05).

These findings should be interpreted with caution, as the observed pattern may reflect complex interrelationships among psychological variables. In multivariable models, suppression effects and intercorrelations between predictors may influence the direction and magnitude of associations [35].

## 4. Discussion

The results of the present study suggest that WRQoL is associated with the psycho-emotional well-being of nurses, as well as with aspects of their sexual health. The findings of the present study demonstrated that depressive symptoms were associated with poorer sexual function, whereas stress showed a positive association with sexual function. Overall, psychological factors emerged as significant predictors of sexual function, highlighting the important role of emotional well-being in women’s sexual health.

### Study Limitations

This research has several limitations that should be considered. First, the non-response rate of 35.5% included both refusal to participate and incomplete questionnaires that were excluded from the analysis. Additional important limitations include the cross-sectional design, the small sample size, and the fact that the study was conducted in a single hospital in Greece, which limits the generalizability of the results and does not allow for causal inferences. The SAW subscale demonstrated low internal consistency (Cronbach’s α = 0.449), which may have affected the reliability of findings involving this variable. Therefore, results related to this subscale should be interpreted with caution. Moreover, the lack of standardized tools in the Greek language for the simultaneous assessment of sexual function in both genders represents a significant methodological limitation. Finally, the possibility of bias due to self-administered questionnaires cannot be excluded, as well as the influence of other unmeasured factors, such as diet, sleep, and exposure to occupational hazards, which may be related to the participants’ mental and sexual health.

The analysis indicated that the level of WRQoL (WRQoL) was moderate to satisfactory, while moderate negative correlations were observed between the total WRQoL score and indicators of depression, anxiety, and stress. These findings are consistent with the study by Barbagianni et al. [36] in Greece, conducted on a sample of 248 healthcare professionals, as well as the study by Kaushik et al. in India [37]. However, the study by Oliveira et al. [38] involving a sample of 4053 nurses in Brazil, although placing the level of WRQoL at moderate levels, conversely revealed moderate to positive correlations with the factors of depression, anxiety, and stress. Multivariate linear regression analysis showed that increased depression, anxiety, and stress were associated with high levels of burnout and secondary traumatic stress. On the other hand, reduced levels of depression, anxiety, and stress were associated with an increase in compassion satisfaction. In relation to these data, the study by Lee in Korea [39] is worth mentioning. This nationwide study included 10,305 nurses, and burnout was assessed at moderate levels. The results revealed a strong positive correlation between stress and burnout. However, the findings documented that the positive aspect of work experiences (compassion satisfaction) can offset the negative aspects (secondary traumatic stress), thereby reducing the level of burnout. WRQoL was positively associated with sexual satisfaction, while no consistent statistically significant associations were observed with other domains of sexual function. These findings can be interpreted within the context of the gender dimension of mental and sexual health. Nevertheless, the study by Kocabaş et al. [40] highlights that shift work negatively affects the sexual function and quality of life of female healthcare workers. In contrast, the study by Andrade-Tasayco in Brazil [41], involving a small sample of 168 female nurses, showed an absence of correlation between dimensions of WRQoL and female sexual dysfunction when adjusted for confounding risk factors. The negative correlation of depression, anxiety, and stress with aspects of sexual function reflects how women experience sexuality under conditions of increased psycho-emotional burden, particularly in high-demand professional environments such as nursing. These findings are consistent with the study by Nülüfer Erbil et al., which demonstrated significant correlations between depression, anxiety, stress, and sexual dysfunction among female nurses. Women working in highly demanding healthcare environments may experience emotional exhaustion, psychological burden, and reduced emotional availability, which can negatively affect intimacy and sexual functioning. The high prevalence of anxiety, depression, and low marital adjustment reported in their study further supports the view that occupational stressors substantially influence women’s sexual and relational well-being [42].

Furthermore, the positive correlation between WRQoL and general well-being with sexual satisfaction suggests that a supportive and healthy work environment may contribute to better mental and sexual health outcomes in women. Lower levels of occupational stress and burnout appear to enhance emotional well-being, intimacy, and sexual functioning. These findings are supported by the systematic review conducted by Priscila Vasconcelos and colleagues, which demonstrated consistent associations between positive sexual health indicators and lower levels of depression and anxiety, as well as higher quality of life and overall well-being across diverse populations. The review emphasized that sexual health is closely interconnected with psychological and emotional health, reinforcing the importance of promoting supportive environments that enhance both well-being and sexual satisfaction [43]. In the present study, stress was positively associated with sexual function in the multivariable model; therefore, this finding should be interpreted with caution. This discrepancy may reflect complex interrelationships among psychological variables; however, further statistical analyses would be required to explore this possibility [33]. Anxiety was not a significant predictor of sexual function in the multivariable model. Previous studies, such as Pham [43], have reported that higher emotional exhaustion is associated with poorer sexual health, which is more consistent with the bivariate findings of the present study than with the regression results.

While there is some evidence that the prevalence of sexual dysfunction decreases as women age [44], other research has concluded that increasing age is associated with a decline in overall physical health and, consequently, sexual function, in connection with chronic diseases such as diabetes and the increasing use of pharmaceutical products [45,46]. In the study by Valenzuela-Peters et al. [47], it is noted that shift work is not a factor associated with sexual dysfunction in female nurses. Instead, low education levels and the presence of chronic disease are identified as burdensome factors for this function. Conversely, hormonal contraception acts as a protective factor.

## 5. Conclusions

In conclusion, the findings suggest that factors such as work–life balance, workplace relationships, and subjective well-being are associated with more favorable outcomes in the mental and sexual health of nurses, although causal relationships cannot be established.

Nevertheless, this study highlights the need for further research to explore the mechanisms linking WRQoL with mental and sexual health. Future studies could include larger samples and multiple hospital settings, employ alternative research methods, and focus on the development and validation of appropriate tools tailored to the Greek context.

## Figures and Tables

**Table 1:** Sociodemographic and Occupational Characteristics of the Sample.

Characteristics	n	%
**Marital/Personal status**		
Single	28	21.7%
In relationship	23	17.8%
Married/Cohabitation	67	51.9%
Divorced	9	7.0%
Widowed	2	1.6%
**Number of children**		
0	61	47.3%
1	20	15.5%
2	40	31.0%
3	7	5.4%
4	1	0.8%
**Educational Level**		
Secondary/Post-Secondary Education	34	26.4%
Higher Education	62	48.1%
Postgraduate Education	33	25.6%
**Annual Income**		
<12.320	52	40.3%
12.320–24.640	69	53.5%
>24.640	8	6.2%
**Work Department**		
Cardiology Departments	7	5.4%
Intensive Care Units (ICU)	37	27.9%
Internal Medicine Department	25	19.4%
Pediatrics Departments	24	18.6%
Outpatient Department	9	7.0%
Emergency Department	15	11.6%
Surgical Department	13	10.1%
**Employment Type**		
Permanent Staff	69	53.5%
Temporary/Contract Staff	60	46.5%
**Desire to Change Department**		
Immediately	14	10.9%
Soon	18	14.0%
In one year	10	7.8%
I Don’t Mind Staying a Few Years	51	39.5%
I Wouldn’t Like to Leave Here	36	27.9%
**Working Hours**		
Fixed	35	27.1%
Rotating Shift	94	72.9%
**Position of Responsibility**		
Yes	38	29.5%
No	91	70.5%
**Diagnosed Conditions**		
Yes	47	36.4%
No	82	63.6%
**Surgeries**		
yes	55	42.6%
no	74	57.4%
**Postmenopausal Estrogen**		
Yes	4	3.1%
No	125	96.9%

Note: For annual income and work department, one response was missing (n = 128).

**Table 2:** Descriptive Statistics of the Study Scales.

	Mean	Std. Deviation	Minimum	Maximum	Cronbach a
FSFI_Desire_Score	3.614	1.387	1.200	6.000	0.930
FSFI_Arousal_Score	4.064	1.720	0.000	6.000	0.974
FSFI_Lubrication_Score	4.348	1.674	0.000	6.000	0.929
FSFI_Orgasm_Score	4.255	1.755	0.000	6.000	0.920
FSFI_Satisfaction_Score	4.508	1.648	0.800	6.000	0.935
FSFI_Pain_Score	4.397	1.883	0.000	6.000	0.955
FSFI_Total_Score	25.489	8.273	2.000	36.000	0.946
DASS_Depression	8.286	8.842	0.000	36.000	0.896
DASS_Anxiety	7.344	7.795	0.000	34.000	0.827
DASS_Stress	12.635	9.744	0.000	42.000	0.900
WRQoL_GWB	3.357	0.744	1.000	5.000	0.817
WRQoL_HWI	3.076	1.020	1.000	5.000	0.792
WRQoL_JCS	3.463	0.714	1.000	5.000	0.763
WRQoL_CAW	3.228	0.864	1.000	5.000	0.794
WRQoL_WCS	2.955	0.874	1.000	5.000	0.761
WRQoL_SAW	2.638	0.901	1.000	5.000	0.449
WRQoL_OVL	2.953	1.030	1.000	5.000	
WRQoL_OVL_Average	3.120	0.609	1.000	4.611	0.851

FSFI = Female Sexual Function Index; DASS = Depression Anxiety Stress Scales; WRQoL = WRQoL; GWB = General Well-Being; HWI = Home–Work Interface; JCS = Job and Career Satisfaction; CAW = Control at Work; WCS = Working Conditions; SAW = Stress at Work; OVL = Overall Quality of Working Life.

**Table 3:** Correlations Between Female Sexual Function Index (FSFI), DASS, and WRQoL Scores.

ψ											
Variable		Desire_	Arousal_	Lubrication_	Orgasm_	Satisfaction_	_Pain_	FSFI_Total_Score	_Depression	_Anxiety	Stress
Depression	Pearson’s r	−0.125	−0.150	−0.098	−0.178 *	−0.302 ***	−0.013	−0.211 *	—		
Anxiety	Pearson’s r	−0.043	−0.067	−0.048	−0.098	−0.192 *	−0.029	−0.128	0.785 ***	—	
Stress	Pearson’s r	−0.004	−0.033	0.010	−0.086	−0.181 *	0.062	−0.094	0.825 ***	0.777 ***	—
GWB	Pearson’s r	0.152	0.161	0.113	0.226 *	0.308 ***	0.071	0.217 *	−0.576 ***	−0.525 ***	−0.588 ***
HWI	Pearson’s r	−0.028	0.021	−0.036	0.083	0.218 *	−0.069	0.067	−0.312 ***	−0.276 **	−0.353 ***
JCS	Pearson’s r	0.092	0.009	−0.069	0.067	0.192 *	−0.062	0.072	−0.313 ***	−0.207 *	−0.234 **
CAW	Pearson’s r	0.058	−0.054	−0.115	−0.045	0.035	−0.093	−0.022	−0.250 **	−0.177 *	−0.155
WCS	Pearson’s r	0.181 *	0.018	−0.041	0.084	0.207 *	−0.026	0.082	−0.203 *	−0.150	−0.282 **
SAW	Pearson’s r	0.090	0.083	0.131	0.141	0.128	0.153	0.139	−0.212 *	−0.297 ***	−0.269 **
OVL	Pearson’s r	0.110	−0.018	−0.076	0.062	0.141	−0.047	0.039	−0.295 ***	−0.249 **	−0.294 ***
OVL_Average	Pearson’s r	0.120	0.052	−0.005	0.127	0.250 **	−0.007	0.125	−0.425 ***	−0.376 ***	−0.436 ***

* *p* < 0.05, ** *p* < 0.01, *** *p* < 0.001. GWB = General Well-Being; HWI = Home–Work Interface; JCS = Job and Career Satisfaction; CAW = Control at Work; WCS = Working Conditions; SAW = Stress at Work; OVL = Overall Quality of Working Life.

**Table 4:** Multiple Linear Regression Analysis Predicting Total FSFI Score.

Predictor	B	SE	β	t	*p*	95% CI for B	Tolerance	VIF
**DASS—Depression**	−0.364	0.164	−0.388	−2.215	0.029	[−0.690, −0.038]	0.212	4.708
**DASS—Anxiety**	0.074	0.163	0.070	0.457	0.648	[−0.248, 0.397]	0.288	3.476
**DASS—Stress**	0.316	0.150	0.371	2.105	0.038	[0.018, 0.614]	0.226	4.424
**WRQoL—General Well-Being (GWB)**	2.066	1.410	0.187	1.465	0.146	[−0.730, 4.862]	0.434	2.303
**WRQoL—Home–Work Interface (HWI)**	1.252	1.062	0.155	1.179	0.241	[−0.853, 3.357]	0.406	2.462
**WRQoL—Job and Career Satisfaction (JCS)**	−0.445	1.911	−0.038	−0.233	0.816	[−4.235, 3.345]	0.264	3.790
**WRQoL—Control at Work (CAW)**	−0.675	1.149	−0.070	−0.588	0.558	[−2.953, 1.603]	0.405	2.469
**WRQoL—Working Conditions (WCS)**	0.261	1.485	0.028	0.176	0.861	[−2.684, 3.206]	0.259	3.858
**WRQoL—Stress at Work (SAW)**	1.122	0.863	0.123	1.300	0.196	[−0.589, 2.832]	0.776	1.289

F = 3.06, (df = 14,103) *p* = 0.001, R^2^ = 29.8% adR^2^ = 18.6%, Standard Error of the Estimate = 7.528. B = unstandardized regression coefficient; SE = standard error; β = standardized regression coefficient; CI = confidence interval. DASS = Depression Anxiety Stress Scales; WRQoL = WRQoL. Model adjusted for age, marital status, number of children, and work experience.

## Data Availability

Data are available from the corresponding author upon reasonable request.
